# Determinants of sputum culture conversion time in multidrug-resistant tuberculosis patients in ALERT comprehensive specialized hospital, Addis Ababa, Ethiopia: A retrospective cohort study

**DOI:** 10.1371/journal.pone.0304507

**Published:** 2024-05-31

**Authors:** Muluye Abebe, Abay Atnafu, Melaku Tilahun, Nejmia Sero, Sebisib Neway, Mekdes Alemu, Getachew Tesfaye, Adane Mihret, Kidist Bobosha, Chengsong Wan

**Affiliations:** 1 School of Public Health, Southern Medical University, Guangzhou, China; 2 Armauer Hansen Research Institute, Addis Ababa, Ethiopia; 3 All Africa Leprosy, Tuberculosis and Rehabilitation Training Center (ALERT) Specialized Hospital, Addis Ababa, Ethiopia; Debre Markos University, ETHIOPIA

## Abstract

**Introduction:**

The treatment response of multi-drug resistance tuberculosis (MDR-Tuberculosis) patients is mainly dictated by the sputum culture conversion. An earlier culture conversion is a remarkable indicator of the improvement in the treatment response. In this study, we aimed to determine the time to culture conversion and its associated factors among MDR-Tuberculosis patients in All Africa Leprosy, Tuberculosis and Rehabilitation Training Center (ALERT) Hospital, Addis Ababa, Ethiopia.

**Methods:**

A retrospective cohort study was conducted on 120 MDR-Tuberculosis patients attending ALERT Hospital from 2018–2022. Kaplan-Meier methods were used to determine the time to initial sputum culture conversion. All relevant laboratory, socio-demographic characteristics, and other clinical data were collected by chart abstraction using a structure data extraction form. The log-rank test was used to determine the survival rate. To identify the predictors of culture conversion, bivariate and multivariate Cox proportional hazard regression analysis was used. The hazard ratio (HR) with a 95% confidence interval was used to estimate the effect of each variable on the initial culture conversion. A test with a P value of < 0.05 was considered statistically significant.

**Results:**

From the total of 120 study participants, 89.2% (107/120) have shown a successful culture conversion. The median age of the participants was 30 years (IQR = 12). The study participants were followed for 408.6 person-months (34.05 person-years). The median time to initial sputum culture conversion was 80 days. The median time to initial sputum culture conversion among HIV-positive and HIV-negative participants was 61 days (IQR = 58–63.5) and 88 days (IQR = 75–91), respectively. HIV-negative and patients with previous treatment history were shown to be the predictor for a prolonged time to initial sputum culture conversion, (aHR = 0.24 (95% CI: 0.1–0.4), P value <0.001) and (aHR = 0.47 (95% CI: 0.31–0.71), P value <0.001) respectively.

**Conclusion:**

The median time to sputum culture conversion for HIV positive was found to be 61 days in our study. Notably, patients with a history of previous anti-tuberculosis treatment, HIV-negative status, and higher bacillary load at baseline exhibited delayed culture conversion. These findings underscore the importance of considering such patient characteristics in the management of MDR-TB cases, as tailored interventions and close monitoring may lead to more favorable treatment outcomes. By identifying individuals with these risk factors early in the treatment process, healthcare providers can implement targeted strategies to optimize patient care and improve overall treatment success rates in MDR-TB management programs.

## Introduction

Tuberculosis (TB) is an infection of the lung and other organs of the body and remains a major public health threat on a global scale [1]. According to the Global Tuberculosis Report 2023, there were an estimated 10.6 million new cases of Tuberculosis worldwide in 2022, with approximately 1.13 million deaths attributed to the disease [2]. Among these cases, it is estimated that 410,000 were multidrug-resistant Tuberculosis (MDR-TB). Ethiopia is classified as one of the 30 countries with a high burden of Tuberculosis and Tuberculosis-HIV co-infection. These figures highlight the significant burden of tuberculosis and drug-resistant tuberculosis in Ethiopia and underscore the importance of continued efforts to strengthen tuberculosis prevention, diagnosis, and treatment initiatives in the country [3].

Ethiopia faces significant challenges in combating MDR-TB, exacerbated by factors such as limited diagnostic capacity, inadequate access to appropriate treatment, and challenges in infection control measures. Despite efforts to improve tuberculosis care, MDR-TB remains a major public health concern, particularly in urban areas where transmission rates are higher. Additionally, co-infection with HIV presents a significant challenge, as it further compromises immune function and increases the risk of treatment failure. The treatment of MDR-TB is usually associated with risks and is expensive, toxic, and in most instances time-consuming as a short treatment regimen in our setting is not fully implemented [4]. Therefore, the follow-up of such MDR-TB patients with culture work-up is robust and requires close monitoring. However, the patient’s infectiousness is determined on the culture result: a conversion from culture positive to negative.

Of note, the goal of infection control measures is reducing the time to culture conversion as MDR-TB patients with culture-positive outcomes are more likely to transmit the infection to others [5, 6]. According to the 2017 WHO guideline, while treating MDR-TB, culture negative results for two consecutive months, usually 30 days apart, is mandatory for concluding culture conversion [7, 8]. Hence, generating evidence regarding the time to culture conversion is not only required for monitoring the treatment success or failure but also to determine when to discontinue the isolation of the MDR-TB patients aiming to reduce the transmission of infection to others.

Clinicians oftentimes rely on culture conversion for the proper management of MDR-TB patients and usually consider it as an efficacy monitoring tool for the treatment response [9]. In this regard, generating evidence about culture conversion becomes inevitably important in making predictions of the time to conversion which eventually helps in planning and determining the overall treatment duration.

On another note, some reports have shown that a time-to-culture conversion is dependent on the host and other host-related factors such as age, comorbidities such as HIV, and Body Mass Index (BMI) [10]. Thus, evidence regarding these factors in association with time-to-culture conversion will have a vital role in the proper management of the patients. In our setting, this phenomenon, a time-to-culture conversion, and its associated factors are not well addressed. Therefore, this study aimed to determine the time to culture conversion and its associated factors among MDR-TB patients in ALERT Hospital, Addis Ababa, Ethiopia.

## Methods

### Study setting, design and period

A retrospective cohort study was conducted on patients attending ALERT Hospital, Addis Ababa, Ethiopia who have a baseline culture positive outcome (bacteriologically confirmed cases) and who were on longer anti- tuberculosis treatment regimen from a period of 2018–2022. ALERT Hospital is one of the largest and oldest hospitals in the country extending its services to a wider community. One of its wards, the MDR-TB ward, provides service to 50–60 MDR-TB patients annually of which 28–32 per year are newly admitted cases.

### Study participants

Multi drug resistant tuberculosis patients enrolled in the MDR TB ward for treatment follow-ups referred from different health facilities.

### Eligibility criteria

Newly enrolled MDR-TB patients who have complete data from culture tests and other relevant clinical data were the study participants. Multi-drug resistant tuberculosis patients with incomplete laboratory data such as those with a follow up period of less than two months, those with a negative culture result at the baseline, and those lacking other relevant clinical data were excluded from the study.

### Sampling technique

The sampling method employed was purposive sampling, encompassing all 120 patients attending MDR TB treatment who were newly admitted and tested positive on smear examination.

### Data collection procedure

All relevant laboratory, socio-demographic characteristics, and other clinical data were collected by chart abstraction using a structure data extraction form. Socio-demographic and laboratory-related data was collected from Armauer Hansen Research Institute (AHRI) tuberculosis laboratory’s registration book recorded from 2018–2022. Armauer Hansen Research Institute’s (AHRI) Tuberculosis (TB) Laboratory provides a routine culture testing and other drug susceptibility testing to MDR patients coming from ALERT MDR ward for the follow up of treatment outcome. The Armauer Hansen Research Institute (AHRI) tuberculosis laboratory holds accreditation as one of Ethiopia’s recognized tuberculosis testing facilities. Apart from offering essential patient services, it also plays a pivotal role in supporting various research endeavors by conducting a diverse range of laboratory procedures. The archived data was accessed on September 26, 2023. All authors had no access to information that could identify individual participants during or after data collection. Other clinical data of the enrolled patient such as medical co-morbidities, history of tuberculosis exposure, previous treatment history, body mass index, and previously used treatment regimen were collected from ALERT’s MDR-TB ward archive and traced using each patient’s medical record number.

### Operational definitions

**Sputum culture conversion time**: refers to the duration of treatment required for a patient with tuberculosis to have negative cultures for *Mycobacterium tuberculosis* in their sputum.

**Sputum culture conversion**: defined as the two consecutive negative cultures separated by at least 30 days. New MDR TB patients was defined as those who have never had treatment for tuberculosis, or have taken anti- tuberculosis drugs for less than 1 month, whereas patients who have taken anti tuberculosis drug for one month or more were defined as previously treated cases [11].

### Data analysis and interpretation

Data was recorded in an Excel spread sheet and was exported to SPSS version 26 for statistical analysis. Descriptive statistics were used to determine the distribution of independent variables and were presented using frequencies, tables, and graphs. Kaplan-Meier methods were used to determine the time to initial sputum culture conversion. The log-rank test was used to determine the survival rate across the strata. To identify the predictors of culture conversion, bivariate and multivariate Cox proportional hazard regression analysis was used. The hazard ratio (HR) with a 95% confidence interval was used to estimate the effect of each variable on the initial culture conversion. A test with a P value of < 0.05 was considered statistically significant.

### Ethical considerations

The study was conducted after obtaining ethical approval (Protocol number: P066/23) from ALERT/AHRI Ethical Review Board, Addis Ababa, Ethiopia. The data was fully anonymized before the data collector has accessed it. The need for consent was waived by the ethics committee.

## Results

### Socio-demographic and clinical characteristics of the study participants

The study included 120 participants with MDR-Tuberculosis, predominantly male (57.5%). The median age was 30, with half falling in the 21–30 age group. Weight loss was reported in 68.3% of participants, while 24.2% had HIV comorbidity. Most participants (62.5%) had a history of previous anti-tuberculosis treatment. Of the total, 23.3% had a baseline smear-negative outcome, and 67.7% had a smear-positive baseline outcome **(Table 1)**.

**Table 1:** Socio-demographic and clinical characteristics of the study participants among multi-drug resistant tuberculosis patients at ALERT comprehensive specialized Hospital from 2018–2022 (N = 120).

Characteristics (N = 120)		Frequency, N (%)
Age group	<20	8 (6.7)
	21–30	60 (50)
	31–40	31 (25.8)
	41–50	12 (10)
	51–60	7 (5.8)
	61–70	2 (1.7)
Sex	Female	51 (42.5)
	Male	69 (57.5)
Weight loss	Yes	82 (68.3)
	No	38 (31.7)
Night sweats	Yes	102 (85)
	No	18 (15)
Comorbidities	Diabetic	10 (8.3)
	Hypertensive	8 (6.7)
HIV	Positive	29 (24.2)
	Negative	91 (75.8)
BMI	Normal	49 (40.8)
	Underweight	71 (59.2)
Previous anti ‐ tuberculosis treatment	New	45 (37.5)
	Previously treated	75 (62.5)
Previously administered anti-tuberculosis treatment regimen	Unknown	7 (9.3)
	First line	64 (85.3)
	Second line	4 (5.3)
AFB smear microscopy	Negative	28 (23.3)
	Scanty	4 (3.3)
	1+	36 (30)
	2+	25 (20.8)
	3+	27 (22.5)

### Initial time to sputum culture conversion

**Fig 1:**
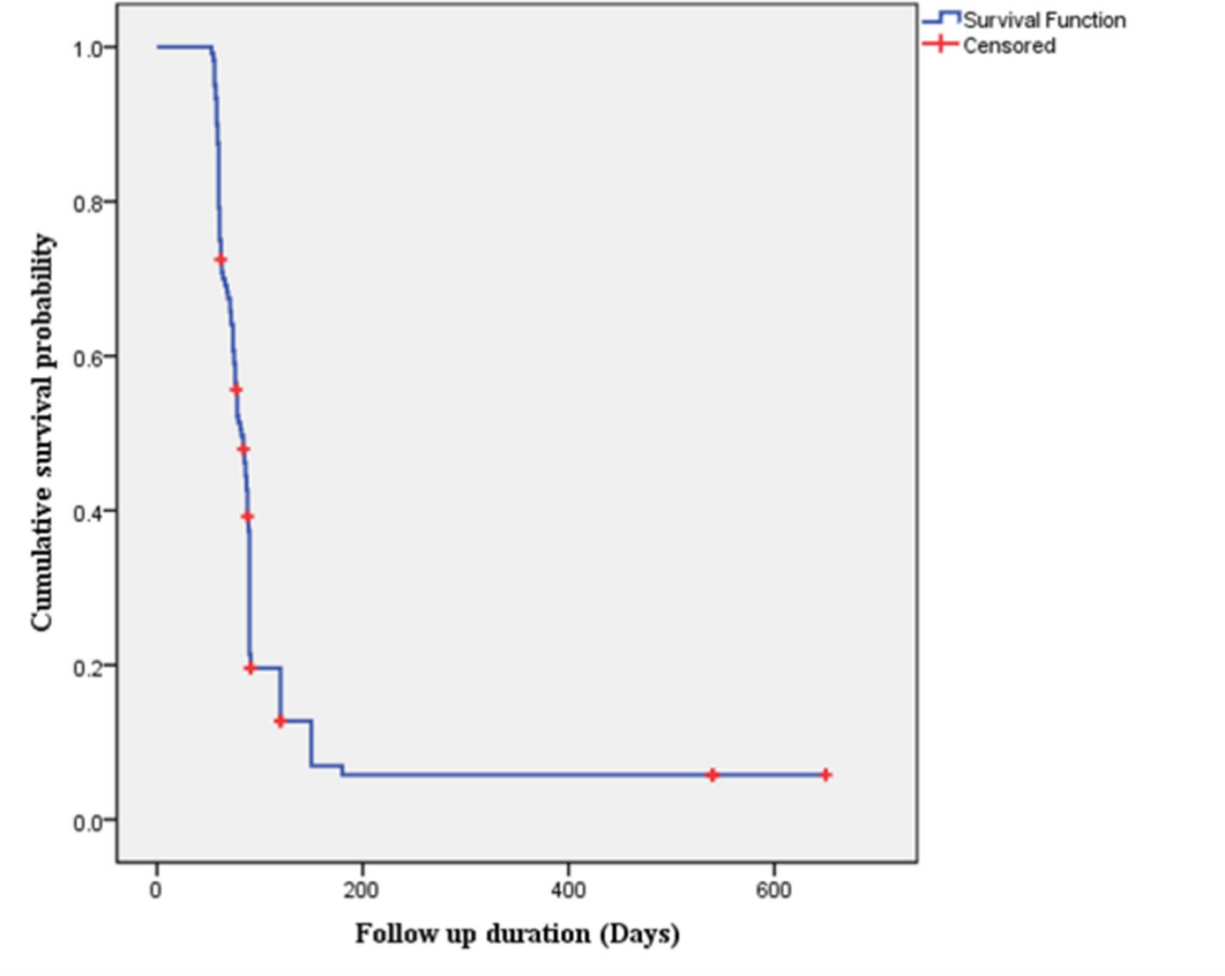
Kaplan Meier survival plot of the initial time to culture conversion among multi-drug resistant tuberculosis patients at ALERT comprehensive specialized hospital, Ethiopia.

Among the participants with a baseline culture-positive outcome, 89.2% (107/120) had a successful culture conversion with a median of 80 days (IQR: 60–90) and 10.8% (13/120) of the participants didn’t have culture conversion. The median time to initial sputum culture conversion among HIV-positive and HIV-negative participants was 61 days (IQR = 58–63.5) and 88 days (IQR = 75–91), respectively. Participants newly introduced to anti-tuberculosis drugs demonstrated a median time to initial sputum culture conversion of 72 days (IQR = 60–84.5). Participants who had a previous anti- tuberculosis treatment history on the other hand have shown a median initial time to sputum culture conversion of 89 days (IQR = 68–120). Participants who had a baseline smear positive outcome with a 3+ grade showed a median initial time to culture conversion of 90 days (IQR = 78–120) (**Fig 1**).

**Fig 2:**
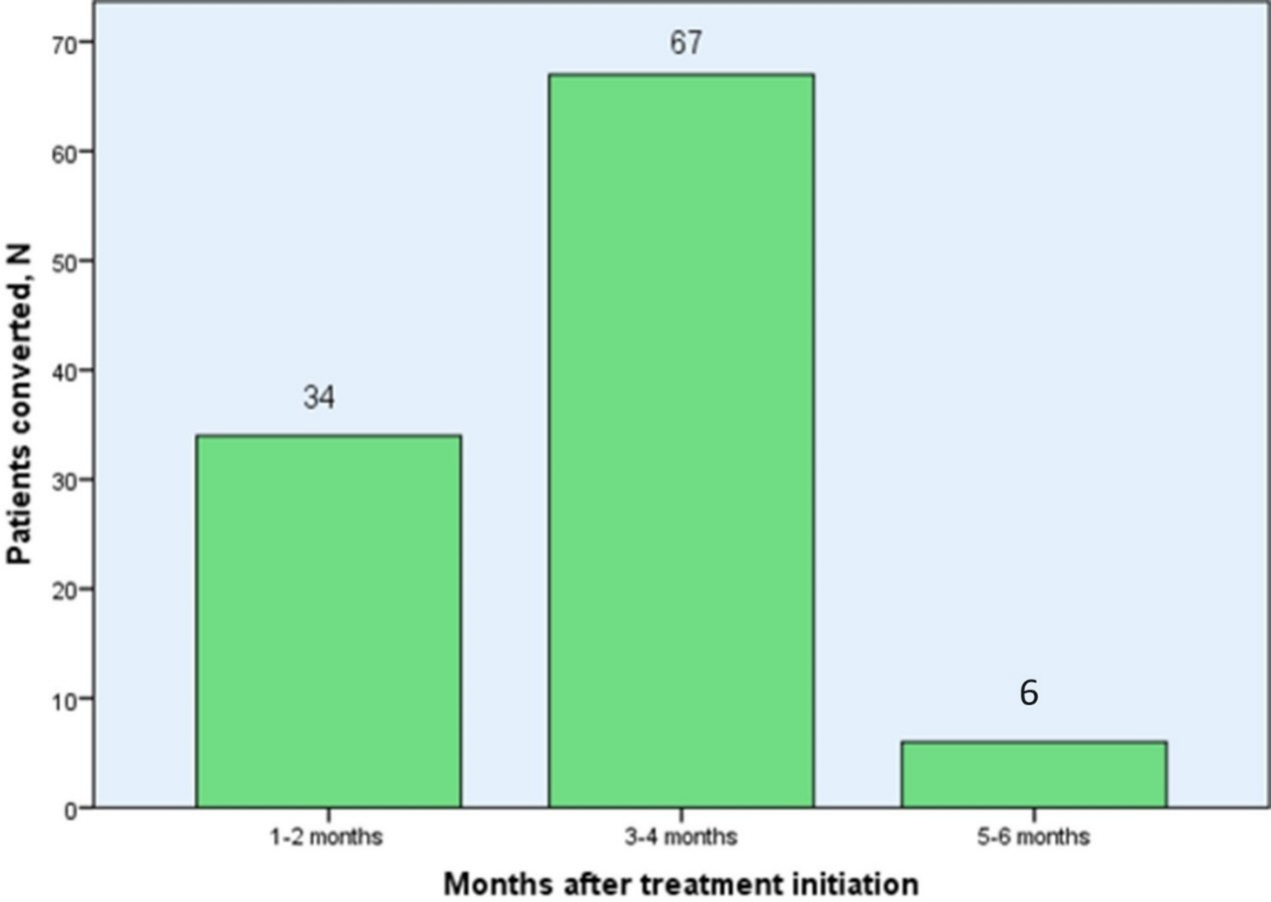
Initial culture conversion of the 107 participants with a culture conversion outcome among multi-drug resistant tuberculosis patients at ALERT comprehensive specialized hospital, Ethiopia.

The study participants were followed for a total of 408.6 person-months (34.05 person-years). More than half, 55.8% (67/120) of the study participants had a sputum culture conversion within three to four months (**Fig 2**).

**Fig 3:**
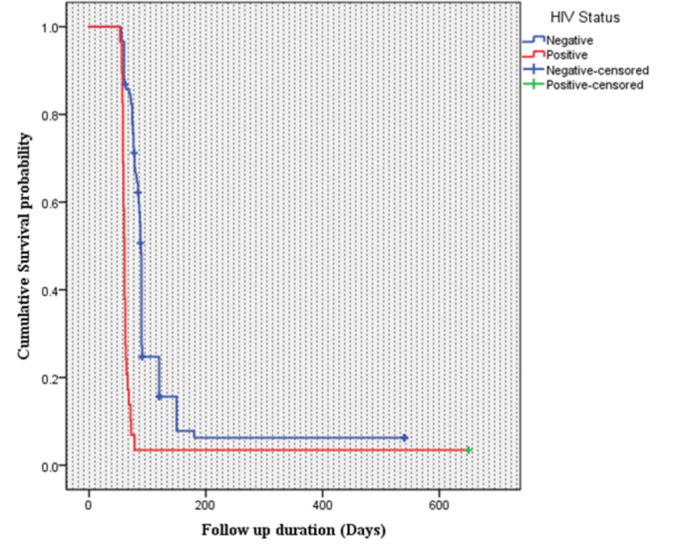
Kaplan Meier survival plot among HIV-positive and HIV-negative individuals at ALERT comprehensive specialized hospital, Ethiopia.

A statistically significant survival distribution, as shown by the log-rank test, has been observed among HIV-positive and HIV-negative participants (X^2^ = 52.7, *P* value < 0.001). Among the HIV-positive individuals, the time to initial sputum culture conversion was rapid when compared with HIV-negative participants (**Fig 3**).

### Determinants of time to culture conversion

A bivariate Cox regression analysis showed that being HIV-negative (cHR = 0.21 (95% CI 0.1–0.3), P value <0.001), previously treatment (cHR = 0.44 (95% CI: 0.3–0.7), P value <0.001), patients with a baseline smear positive with 1+ (cHR = 0.6 (95% CI: 0.36–1.0), P value = 0.03), 2+ (cHR = 0.45 (95% CI: 0.25–0.8), P value = 0.009) & 3+ (cHR = 0.4 (95% CI: 0.23–0.7), P value = 0.001) grades were significant predictors for the delay in the initial sputum culture conversion **(Table 2)**.

**Table 2:** Bivariate and multivariate Cox logistic regression analysis of the predictor to initial sputum culture conversion from MDR Tuberculosis patients at ALERT comprehensive specialized Hospital, Ethiopia.

Characteristics (N = 120)		Sputum culture conversion status		Total	cHR (95% CI)	*P* value	aHR (95% CI)	*P* value
		Event (N = 107)	Censored (N = 13)					
Age group	>20	7	1	8	Ref			
	21–30	52	8	60	2.36 (0.48–11.5)	0.29		
	31–40	27	4	32	1.23 (0.3–5.2)	0.76		
	41–50	12	0	12	1.19 (0.28–5.0)	0.81		
	51–60	7	0	7	1.76 (0.39–7.9)	0.46		
	61–70	2	0	2	1.8 (0.37–8.8)	0.45		
Sex	Female	44	7	51	Ref			
	Male	63	6	69	1.07 (0.73–1.58)	0.7		
Night sweats	Yes	91	11	102	Ref			
	No	16	2	18	1.07 (0.63–1.8)	0.8		
HIV	Positive	28	1	29	Ref		Ref	
	Negative	79	12	91	0.21 (0.1–0.3)	0.000*	0.24 (0.1–0.4)	0.000*
BMI	Normal	47	2	49	Ref			
	Underweight	60	11	71	1.4 (0.95–2.07)	0.08		
Previous anti- tuberculosis treatment	No	64	11	45	Ref		Ref	
	Yes	43	2	75	0.44 (0.3–0.7)	0.000*	0.47 (0.31–0.71)	0.000*
AFB smear microscopy	Negative	28	0	28	Ref		Ref	
	Scanty	4	0	4	0.97 (0.34–2.8)	0.9	1.1 (0.4–3.3)	0.8
	1+	33	3	36	0.6 (0.36–1.0)	0.04*	0.94 (0.54–1.6)	0.8
	2+	18	7	25	0.45 (0.25–0.8)	0.009*	0.78 (0.4–1.5)	0.46
	3+	24	3	27	0.4 (0.23–0.7)	0.001*	0.53(0.29–0.9)	0.03*
Previously administered anti-tuberculosis treatment regimen	First line	56	8	64	0.58 (0.16–2.0)	0.4		
	Second line	3	1	4	0.25 (0.02–2.9)	0.27		
	unknown	48	4	52	Ref			

The multivariate Cox regression analysis also showed that being HIV-negative (aHR = 0.24 (95% CI: 0.1–0.4), P value <0.001), previous treatment (aHR = 0.47 (95% CI: 0.31–0.71), P value <0.001), & patients with a baseline smear positive with 3+ grade (aHR = 0.53 (95% CI:0.29–0.9), P value = 0.03) were statistically significant predictor for prolonged time to initial sputum culture conversion.**(Table 2)**.

## Discussion

The timeframe necessary for sputum culture conversion following the initiation of treatment serves as a crucial indicator of treatment effectiveness among patients with multidrug-resistant or rifampicin-resistant tuberculosis (MDR/RR-Tuberculosis) undergoing anti-tuberculosis therapy [12–14]. In our investigation, we focused on MDR-Tuberculosis patients with initial positive baseline cultures, with a median age of 30 years. Similar findings have been reported in previous studies conducted in Ethiopia [12, 15]. Similar findings have been reported in previous studies conducted in Ethiopia. Among our study participants, we observed a 24.2% prevalence of HIV co-morbidity. Conversely, a study conducted in the Tigray region of Ethiopia reported a lower rate of HIV co-morbidity [16]. This disparity could potentially be attributed to the higher prevalence of HIV in Addis Ababa compared to other regions [17].

One challenge in managing MDR-Tuberculosis patients is the heightened risk of relapse and treatment failure following standard first-line anti-tuberculosis drug completion, particularly in settings with limited universal Drug Susceptibility Testing (DST) coverage. The prevalence of such events is accentuated by the scarcity of culture and DST laboratories, which are insufficient for a population of over 110 million. To address this challenge, expanding services across all regions becomes imperative [18]. In our study, 62.5% of the participants were previously treated with standard first-line anti-tuberculosis drugs. Similarly, a higher rate of MDR-Tuberculosis patients with a previous treatment history has been reported [15, 19].

The duration for culture conversion, as determined by the median, was observed to be 80 days in our study, and similar reports with a median culture conversion time of 85 days and 83 days were reported in China and Latvia respectively [14, 20]. On another note, a relatively lower rate of culture conversion median time was reported in Pakistan, Peru, South Africa, and some regions of Ethiopia [10, 12, 13, 15, 21, 22]. Our result showed a relatively higher culture conversion median time, possibly due to the small sample size employed in our study.

The time taken to achieve a successful sputum culture conversion can be influenced by various factors, including the treatment regimens used, the susceptibility of second-line drugs, the rate of loss to follow-up, and the sensitivity of laboratory testing [23]. Our study showed a higher culture conversion time of 89.2%. A comparable rate of successful culture conversion rate, 85%, was shown by a study from Ethiopia [24]. Similarly, a comparable findings of the culture conversion rates reported from studies conducted in Hangzhou, China 93.5% [14], Peru 87.7% [21], Latvia 84% [20], Pakistan 76.6% [10], Northwest Ethiopia 61.7%-88.6% [13], Amhara region Ethiopia 86.7% [15], Tigray region, Ethiopia 91% [12]. The observed higher culture conversion rate of 89.2% in our study is comparable with the above reports and could be attributed to the rigorous adherence to the standard drug regimen and the meticulous follow-up of MDR-Tuberculosis patients at the center. However, a comprehensive investigation is necessary to pinpoint and comprehend the specific factors contributing to this outcome, such as treatment regimens, regional influences, loss to follow-up dynamics, laboratory testing variations, and individual patient characteristics. Identifying these shades is crucial for refining treatment strategies and enhancing overall success rates.

A higher rate of sputum culture conversion was observed among participants with HIV-positive MDR-Tuberculosis patients as compared to HIV-negative MDR-Tuberculosis patients in our study. Reports consistent with our finding, showing the higher rate of culture conversion among HIV-positive MDR-Tuberculosis patients have been shown by some studies [12, 13, 21]. However, reported by other studies have shown inconsistent findings with the results reported in this study [25–28]. The reason for achieving an early culture conversion among HIV-positive participants might be due to an effective Tuberculosis-HIV integrated work, possibly indicating good adherence to the use of anti-tuberculosis drugs among HIV-positive cases. In addition, this variance could potentially be attributed to factors such as the stage of AIDS, adherence to antiretroviral therapy (ART), and nutritional status. However, conducting further research at a larger scale may help comprehend the identification process of the factors related to such events. On another note, we have observed in our study that patients without a previous history of anti-tuberculosis treatment have shown a rapid culture conversion rate when compared with those with a previous treatment history. This indicates that exposure to anti-tuberculosis treatment might be one of the determinant factors of culture conversion. This was evidenced in our study in that the previously treated participants showed a statistically significant association with a lower rate of culture conversion, (cHR = 0.44 (95% CI: 0.3–0.7), *P* value <0.001) and (aHR = 0.47 (0.31–0.71), *P* value <0.001). Kurbatova et al have shown a finding consistent with our results [29]. Similarly, consistent findings have been shown by Tierney et al [21]. In addition, the presence of acid-fast bacilli (AFB) microscopy grade 2+ and 3+ and being HIV negative were identified as a statistically significant predictor for an extended period required for the initial sputum culture conversion in our study. Similar observations have been shown in previous studies conducted elsewhere [12, 15]. The reason for slower conversion might be that HIV-negative individuals generally may have a more robust immune response compared to those with HIV, which could potentially lead to a slower clearance of mycobacterial infection and hence delayed sputum culture conversion. Additionally, previous treatment history suggests that these individuals may have been exposed to anti-Tuberculosis drugs before, possibly leading to the development of drug resistance and thereby complicating treatment outcomes. Furthermore, previous treatment may have resulted in selecting more resistant strains of Mycobacterium tuberculosis, making them less susceptible to standard anti-Tuberculosis medications and prolonging the time to sputum culture conversion. Overall, these factors highlight the importance of considering HIV status and treatment history when predicting the time to sputum culture conversion in MDR-Tuberculosis patients.

Due to lack of a complete set of laboratory and clinical data, several patients were excluded from this study that limited the sample size. The limited sample size in our study may impact the generalizability of the findings and reduce the statistical power of the study, potentially affecting the ability to detect significant associations or differences. Additionally, the retrospective nature of the study design may introduce biases related to data completeness. Despite these limitations, the study provides valuable insights into the time to sputum culture conversion among MDR TB patients, but cautious interpretation is warranted.

## Conclusion

In our study, we observed successful sputum culture conversion within the first six months for the majority of participants. Notably, over half of the participants achieved conversion within the initial 3–4 months. The median time to culture conversion in our study was found to be lower than the WHO recommendation. Our findings underscore the importance of diligent management of MDR-Tuberculosis patients, particularly those with a history of previous anti-tuberculosis treatment, HIV-negative status, and higher bacillary load at baseline. We observed delayed culture conversion and a significant association with a lower sputum culture conversion rate in these patient subgroups, highlighting the need for tailored treatment approaches and close monitoring.

## Supporting information

S1 Dataset (SAV)

## References

[1] Natarajan A, Beena PM, Devnikar AV, Mali S. A systemic review on tuberculosis. The Indian journal of tuberculosis. 2020;67(3):295–311. doi: 10.1016/j.ijtb.2020.02.005 32825856

[2] WHO. Global TUberculosis Report. 2023.

[3] Bagcchi SJTLM. WHO’s global tuberculosis report 2022. 2023;4(1):e20. 10.1016/S2666-5247(22)00359-7 36521512

[4] Vanino E, Granozzi B, Akkerman OW, Munoz-Torrico M, Palmieri F, Seaworth B, et al. Update of drug-resistant tuberculosis treatment guidelines: A turning point. International journal of infectious diseases: IJID: official publication of the International Society for Infectious Diseases. 2023;130 Suppl 1:S12-s5. doi: 10.1016/j.ijid.2023.03.013 36918080

[5] Assemie MA, Alene M, Petrucka P, Leshargie CT, Ketema DB. Time to sputum culture conversion and its associated factors among multidrug-resistant tuberculosis patients in Eastern Africa: A systematic review and meta-analysis. International Journal of Infectious Diseases. 2020;98:230–6. doi: 10.1016/j.ijid.2020.06.029 32535296

[6] WHO. WHO consolidated guidelines on drug-resistant tuberculosis treatment: World Health Organization; 2019. 30946559

[7] WHO. Guideline for Treatment of Drug Susceptible Tuberculosis and Patient Care. 2017.

[8] Kurbatova EV, Cegielski JP, Lienhardt C, Akksilp R, Bayona J, Becerra MC, et al. Sputum culture conversion as a prognostic marker for end-of-treatment outcome in patients with multidrug-resistant tuberculosis: a secondary analysis of data from two observational cohort studies. The Lancet Respiratory medicine. 2015;3(3):201–9. doi: 10.1016/S2213-2600(15)00036-3 25726085 PMC4401426

[9] Martin MK, Paul OJ, Sara R, Hilary A, Frank M, Augustin MK, et al. High rates of culture conversion and low loss to follow-up in MDR-TB patients managed at Regional Referral Hospitals in Uganda. BMC Infectious Diseases. 2021;21(1):1060. doi: 10.1186/s12879-021-06743-y 34641816 PMC8507334

[10] Iqbal Z, Khan MA, Aziz A, Nasir SM. Time for culture conversion and its associated factors in multidrug-resistant tuberculosis patients at a tertiary level hospital in Peshawar, Pakistan. Pak J Med Sci. 2022;38(4Part-II):1009–15. 35634598 10.12669/pjms.38.4.5058 PMC9121929

[11] WHO, Initiative ST. Treatment of tuberculosis: guidelines: World Health Organization; 2010. 23741786

[12] Weldemhret L, Atsbaha AH, Bekuretsion H, Desta A, Legesse L, Kahsay AG, et al. Time to Sputum Culture Conversion and Its Predictors Among Multidrug Resistant Tuberculosis Patients in Tigray, Northern Ethiopia: Retrospective Cohort Study. Infection and Drug Resistance. 2023;Volume 16:3671–81. doi: 10.2147/IDR.S413495 37324659 PMC10263018

[13] Shibabaw A, Gelaw B, Wang SH, Tessema B. Time to sputum smear and culture conversions in multidrug resistant tuberculosis at University of Gondar Hospital, Northwest Ethiopia. PLoS One. 2018;13(6):e0198080. doi: 10.1371/journal.pone.0198080 29944658 PMC6019386

[14] Li Q, Lu M, Hsieh E, Wu L, Wu Y, Wang M, et al. Time to sputum culture conversion and its predictors among patients with multidrug-resistant tuberculosis in Hangzhou, China: A retrospective cohort study. Medicine (Baltimore). 2020;99(50):e23649. doi: 10.1097/MD.0000000000023649 33327347 PMC7738096

[15] Yihunie Akalu T, Muchie KF, Alemu Gelaye K. Time to sputum culture conversion and its determinants among Multi-drug resistant Tuberculosis patients at public hospitals of the Amhara Regional State: A multicenter retrospective follow up study. PloS one. 2018;13(6):e0199320. doi: 10.1371/journal.pone.0199320 29927980 PMC6013102

[16] Weldemhret L, Atsbaha AH, Bekuretsion H, Desta A, Legesse L, Kahsay AG, et al. Time to Sputum Culture Conversion and Its Predictors Among Multidrug Resistant Tuberculosis Patients in Tigray, Northern Ethiopia: Retrospective Cohort Study. Infection and drug resistance. 2023;16:3671–81. doi: 10.2147/IDR.S413495 37324659 PMC10263018

[17] Kibret GD, Ferede A, Leshargie CT, Wagnew F, Ketema DB, Alebel A. Trends and spatial distributions of HIV prevalence in Ethiopia. Infectious diseases of poverty. 2019;8(1):90. doi: 10.1186/s40249-019-0594-9 31623689 PMC6796490

[18] WHO. Treatment of tuberculosis: guidelines for national programmes. Treatment of tuberculosis: guidelines for national programmes1997. p. 77-.

[19] Alene KA, Viney K, Yi H, McBryde ES, Yang K, Bai L, et al. Comparison of the validity of smear and culture conversion as a prognostic marker of treatment outcome in patients with multidrug-resistant tuberculosis. PloS one. 2018;13(5):e0197880. doi: 10.1371/journal.pone.0197880 29791488 PMC5965863

[20] Timothy H. Holtz M MPH, Maya Sternberg PhD, Steve Kammerer MBA, Kayla F. LasersonScD;, Vija Riekstina MD, Evija Zarovska MD; et al. <Time to sputum culture conversion in multidrug-resistant tuberculosis predictors and relationship to treatment outcome.pdf>. Annals of Internal Medicine. May 02,2006;Volume 144 •. 10.7326/0003-4819-144-9-200605020-00008 16670134

[21] Tierney DB, Franke MF, Becerra MC, Alcántara Virú FA, Bonilla CA, Sánchez E, et al. Time to culture conversion and regimen composition in multidrug-resistant tuberculosis treatment. PloS one. 2014;9(9):e108035. doi: 10.1371/journal.pone.0108035 25238411 PMC4169600

[22] Ncha R, Variava E, Otwombe K, Kawonga M, Martinson NA. Predictors of time to sputum culture conversion in multi-drug-resistant tuberculosis and extensively drug-resistant tuberculosis in patients at Tshepong-Klerksdorp Hospital. S Afr J Infect Dis. 2019;34(1):111. doi: 10.4102/sajid.v34i1.111 34485452 PMC8377786

[23] Musteikienė G, Miliauskas S, Zaveckienė J, Žemaitis M, Vitkauskienė A. Factors associated with sputum culture conversion in patients with pulmonary tuberculosis. Medicina. 2017;53(6):386–93. doi: 10.1016/j.medici.2018.01.005 29496377

[24] Shibabaw A, Gelaw B, Wang S-H, Tessema B. Time to sputum smear and culture conversions in multidrug resistant tuberculosis at University of Gondar Hospital, Northwest Ethiopia. PloS one. 2018;13(6):e0198080. doi: 10.1371/journal.pone.0198080 29944658 PMC6019386

[25] Russkikh A, Korotych O, Sereda Y, Samoilova A, Achar J, Yedilbayev A, et al. Factors associated with culture conversion among adults treated for pulmonary extensively drug-resistant tuberculosis during 2018–2019 in the Russian Federation: an observational cohort study. Monaldi Arch Chest Dis. 2021;91(1). doi: 10.4081/monaldi.2021.1678 33470087

[26] Rieu R, Chang C, Collin SM, Fazekas J, Dassanaike S, Abbara A, et al. Time to detection in liquid culture of sputum in pulmonary MDR-TB does not predict culture conversion for early discharge. J Antimicrob Chemother. 2016;71(3):803–6. doi: 10.1093/jac/dkv407 26661394

[27] Velayutham B, Nair D, Kannan T, Padmapriyadarsini C, Sachdeva KS, Bency J, et al. Factors associated with sputum culture conversion in multidrug-resistant pulmonary tuberculosis. (1815–7920 (Electronic)). 10.5588/ijtld.16.0096 27931345

[28] Pai M, Brust JCM, Lygizos M, Chaiyachati K, Scott M, van der Merwe TL, et al. Culture Conversion Among HIV Co-Infected Multidrug-Resistant Tuberculosis Patients in Tugela Ferry, South Africa. PLoS ONE. 2011;6(1). 10.1371/journal.pone.0015841 PMC3017058 21253585

[29] Kurbatova EV, Gammino VM, Bayona J, Becerra MC, Danilovitz M, Falzon D, et al. Predictors of sputum culture conversion among patients treated for multidrug-resistant tuberculosis. The international journal of tuberculosis and lung disease: the official journal of the International Union against Tuberculosis and Lung Disease. 2012;16(10):1335–43. doi: 10.5588/ijtld.11.0811 23107633

